# Massively parallel multiplex DNA sequencing for specimen identification using an Illumina MiSeq platform

**DOI:** 10.1038/srep09687

**Published:** 2015-04-17

**Authors:** Shadi Shokralla, Teresita M. Porter, Joel F. Gibson, Rafal Dobosz, Daniel H. Janzen, Winnie Hallwachs, G. Brian Golding, Mehrdad Hajibabaei

**Affiliations:** 1 Department of Integrative Biology and Biodiversity Institute of Ontario, University of Guelph, 50 Stone Road East, Guelph, ON, Canada N1G 2W1; 2 Department of Biology, McMaster University, 1280 Main Street West, Hamilton, ON, Canada L8S 4K1; 3 Department of Biology, University of Pennsylvania, Philadelphia, PA, USA 19104

## Abstract

Genetic information is a valuable component of biosystematics, especially specimen identification through the use of species-specific DNA barcodes. Although many genomics applications have shifted to High-Throughput Sequencing (HTS) or Next-Generation Sequencing (NGS) technologies, sample identification (e.g., via DNA barcoding) is still most often done with Sanger sequencing. Here, we present a scalable double dual-indexing approach using an Illumina Miseq platform to sequence DNA barcode markers. We achieved 97.3% success by using half of an Illumina Miseq flowcell to obtain 658 base pairs of the cytochrome *c* oxidase I DNA barcode in 1,010 specimens from eleven orders of arthropods. Our approach recovers a greater proportion of DNA barcode sequences from individuals than does conventional Sanger sequencing, while at the same time reducing both per specimen costs and labor time by nearly 80%. In addition, the use of HTS allows the recovery of multiple sequences per specimen, for deeper analysis of genetic variation in target gene regions.

The use of DNA sequences in biosystematics has revolutionized our understanding of biodiversity from elucidating deep branches of the Tree of Life to exploring species boundaries and population-level variations in communities and ecosystems. For example, short, standardized species-specific DNA sequences - DNA barcodes - have been demonstrated to work well for specimen identification in systematics^1^,^2^, ecological research^3^, biodiversity inventories^4^,^5^, museum collection research^6^, and forensic applications^7^. Target gene regions have been established as DNA barcodes for each kingdom of life (e.g., cytochrome oxidase *c* subunit I (*COI*) for animals^8^, nuclear internal transcribed spacer (*ITS*) for fungi^9^, and *rbcL* and *matK* chloroplast genes for plants^10^). A number of initiatives currently seek to build and curate public DNA barcode databases for the purpose of recording, counting, and identifying global biodiversity^11^,^12^,^13^,^14^. In order for DNA sequence libraries to be of maximal value, they must be built so as to represent major amounts of the described and undescribed global diversity^15^,^16^,^17^.

Type specimens for each species - holotypes - are by definition, the reference for a given species. It has been suggested that DNA barcode data for these holotype specimens are necessary for databases^18^. Many of these type specimens are contained in museum collections, are very old, and are generally unavailable for standard genomic data gathering^6^. Special protocols are needed to access the massive potential sources of data presently stored in natural history collections.

Another major source of specimens for DNA barcode-based studies is mixed environmental samples. These samples come from Malaise traps^19^, freshwater and marine benthos^20^,^21^, meiofauna^22^, and marine zooplankton^23^. From the arctic to the neotropics, such mixed environmental samples have revealed a high degree of species-level genetic diversity^5^. The recovery of DNA sequence data from both museum specimens and bulk-collected environmental samples will greatly facilitate the construction of DNA barcode libraries.

Conventional PCR amplification followed by dideoxy chain-termination sequencing (also known as Sanger sequencing^24^) has been used for the production of nearly all of the existing content of public DNA barcode libraries. Cost limitations of Sanger sequencing per specimen, however, severely restrict its ability to be scaled up to deal with millions of specimens requiring DNA barcoding. In addition, Sanger sequencing requires relatively high concentrations of high quality DNA template in order to be successful^25^. Even when successful, the process produces only a single sequencing signal pattern, or electropherogram, of a maximum of 1,500 base pairs per individual. This single sequence can be the product of co-amplification of other DNA templates present with the target individual (e.g., intrasample contamination, *Wolbachia* infection, gut contents) and may not represent the ‘true’ genetic marker of the target individual^26^. This case is common when attempting to recover DNA sequence data from individuals isolated from bulk mixed samples (e.g., Malaise traps, benthic samples, soil meiofauna, marine zoo- and phytoplankton). These circumstances can introduce intra-sample contamination and it is often necessary to use vector-based cloning or gel excision to be able to recover the target gene sequence. These methods are time consuming and labor-intensive. Another challenge in recovering DNA sequence data from an individual is specimen body size for some groups. The meiofauna represent organisms from all branches of Animalia that fall roughly between 50 μm and 0.5 mm in size^20^. Due to their size, meiofaunal organisms cannot be reliably tissue subsampled or, in some cases isolated individually. This restriction has severely hampered efforts to generate genetic marker libraries for these important groups.

We have developed a new multiplexing approach to recovering DNA barcode sequences from individuals that addresses the problem of isolating individuals from mixed environmental samples. By utilizing the high throughput sequencing power of Illumina MiSeq, a platform with a relatively small size and lower operating cost, we generate a large number of full-length (658 bp) DNA barcode sequences from a diverse group of organisms in a single sequencing run. We sequenced and assembled two smaller overlapping fragments of the *COI* barcode region to overcome the primer specificity challenges for the recovery of DNA barcodes. Increased sequencing depth per specimen allowed for the generation of multiple DNA sequences per specimen. Bioinformatic analysis of these sequences determined the ‘true’ barcode for an individual and distinguished it from likely intra-sample contamination, *Wolbachia*, pseudogenes, or other intrusions. While we used *COI* sequences, this method is adaptable to any chosen genetic marker. It is also scalable to thousands of individuals per sequencing run. Not only did this method recover a greater proportion of DNA sequence data from individuals than did conventional Sanger sequencing, it also produced it at a much lower cost per specimen.

## Results

A total of 1,010 individual arthropods were isolated from a single Malaise trap sample from Area de Conservación Guanacaste, northwestern Costa Rica. Each individual was isolated, morphologically identified to order, and tissue subsampled. Tissue subsamples (i.e., a leg from each individual) were separated into eleven 96-well tissue plates and DNA extracted.

**Figure 1:**
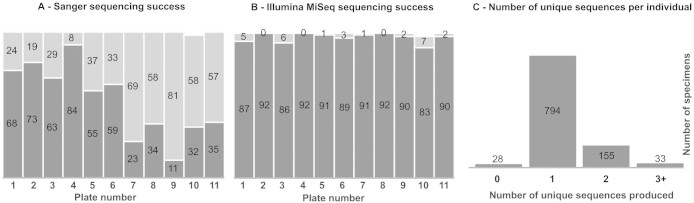
Results of both Sanger and Illumina MiSeq sequencing of 1,010 individual arthropods from a single Malaise trap sample. (A) Overall success of generating *COI* DNA sequences via Sanger sequencing for each of eleven 96-well specimen plates. (B) Overall success of generating *COI* DNA sequences via Illumina MiSeq sequencing for each of eleven plates. For (A) and (B), number of individuals per plate producing a *COI* sequence are shaded dark below, with unsuccessful individuals above. (C) Number of unique *COI* DNA sequences produced via Illumina MiSeq sequencing for each individual.

The standard 5′ end of the *COI* region was amplified for each individual DNA template using the primers LCO1490 and HCO2198^27^. These amplicons were sequenced via standard Sanger protocols. A total of 537 individuals (53.2%) produced a full-length (>500 bp) sequence via Sanger sequencing (Fig. 1A). Sanger sequencing success ranged from 12.0% (plate 9) up to 91.3% (plate 4). A total of 983 individuals (97.3%) produced at least one full-length sequence via Illumina MiSeq sequencing (Fig. 1B). The same region of *COI* was amplified for all individual DNA templates in two smaller, overlapping fragments using Ill_LCO1490 x Ill_C_R and Ill_B_F x Ill_HCO2198 primer sets respectively. The two fragments overlap by 82 bp. All generated amplicons were dual indexed with unique 5-mer multiple identifiers (MIDs) from both directions. The generated amplicons were pooled in groups and re-dual indexed and sequenced on half of a single Illumina MiSeq flowcell using a V3 Miseq sequencing kit (300 bp × 2). A total of 18,873,718 Illumina paired-end reads were filtered for quality and length. Across each of the eleven 96-well plates, a total of 10,480,349 raw FC fragment reads were Illumina paired-end sequenced (mean - 952,759 reads per plate) and a total of 8,393,369 raw BR fragment reads were sequenced (mean - 763,034 reads per plate). For each of the eleven plates, the raw paired-end reads for the FC fragment and, separately for the BR fragment, were merged with a minimum overlap of 25 bp. A total of 9,652,825 paired FC reads (mean - 877,530 paired reads per plate) and a total of 6,020,424 paired BR reads (mean 547,311 paired reads per plate) were retained for further processing. After MID sorting and primer trimming, putative chimeric sequences were removed along with identical duplicate sequences using a 99% sequence similarity cutoff. The two fragments of each individual were paired, requiring a minimum of 80 bp overlap; a maximum of 0.02 (2%) mismatches were allowed in the overlap region. An average of 5,868 (range 5,166 – 6,577) full-length sequences were produced for each individual. Following de-replication of identical sequences, the number of unique, abundant sequences (>10% of total sequences per individual) recovered for each individual ranged from zero to six. Illumina MiSeq sequencing success ranged from 92.2% (plate 10) up to 100% (plates 2, 4, and 8). A total of 794 individuals (78.6%) produced exactly one unique full-length assembled *COI* sequence via Illumina MiSeq sequencing (Fig. 1C).

**Figure 2:**
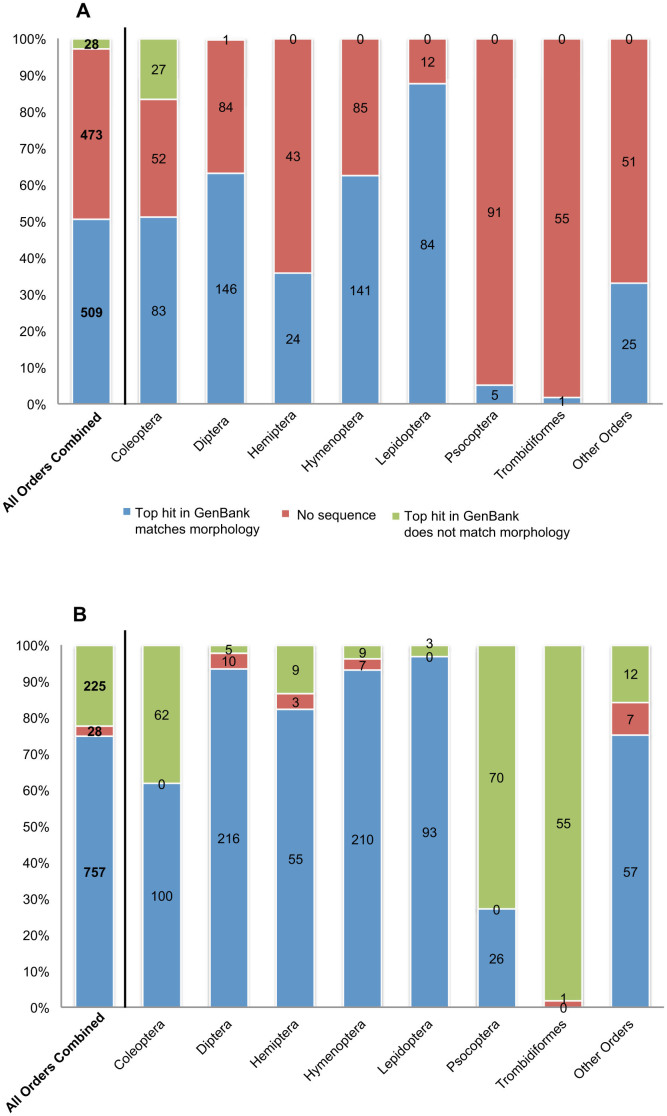
Number and percentage of 1,010 individual arthropod specimens producing a *COI* DNA sequence that matches morphological identification based on BLAST comparison to public DNA barcode databases. Panel (A) Sanger-generated barcodes. Panel (B) Illumina-generated barcodes.

All sequences produced by both Sanger and Illumina MiSeq sequencing were identified via BLAST^28^ comparison to public *COI* databases. Each top hit BLAST result for each sequence for each individual was then compared to morphological identification (Fig. 2). A total of 509 individuals (50.4%) produced a DNA sequence matching morphological identification via Sanger sequencing. The number of non-matching sequences was 28 (2.8%), with the remaining 473 individuals (46.8%) producing no Sanger sequence at all. The percentage of matching Sanger sequences differed between arthropod orders, ranging from 1.8% for Trombidiformes to 62.4% for Hymenoptera, 63.2% for Diptera and 87.5% for Lepidoptera (Fig. 2A).

A total of 757 individuals (75.0%) produced a *COI* sequence matching morphological identification via Illumina MiSeq sequencing. The number of non-matching sequences was 225 (22.3%), with the remaining 27 individuals (2.7%) producing no Illumina MiSeq sequence at all. The percentage of matching Illumina MiSeq sequences differed between arthropod orders, ranging from 0.0% for Trombidiformes to 92.9% for Hymenoptera, 93.5% for Diptera and 96.9% for Lepidoptera (Fig. 2B).

**Figure 3:**
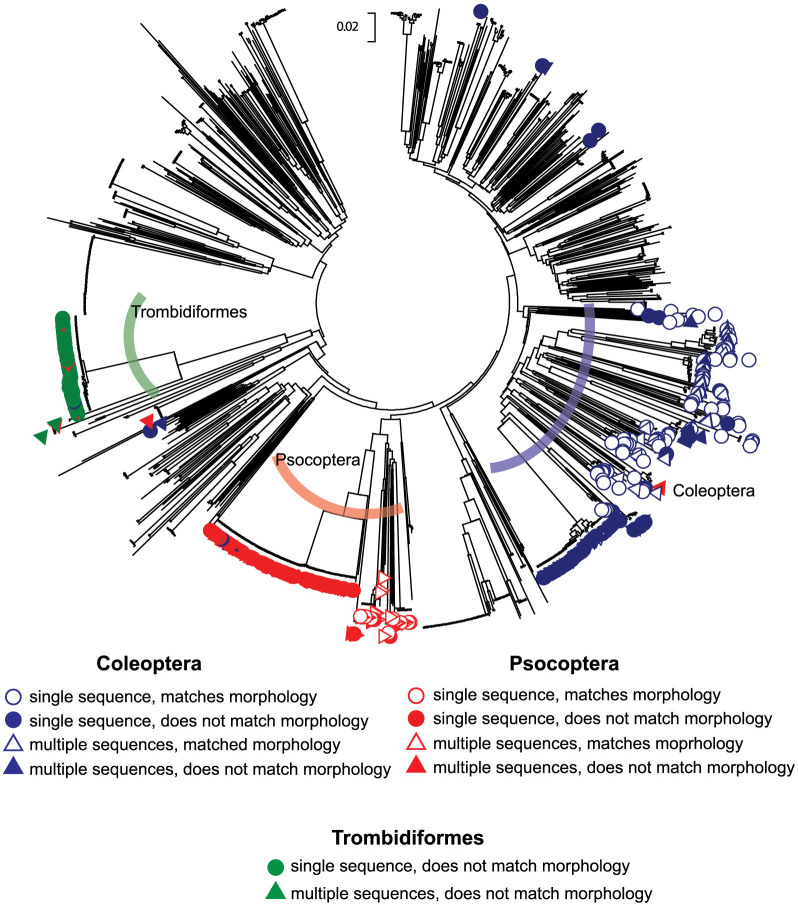
Neighbor-joining diagram of 1,211 *COI* sequences produced from Illumina MiSeq sequencing of 1,010 individual arthropods. Distance measurement is calculated in number of base substitutions per site based on the Kimura 2-parameter method. Sequences originating from individuals morphologically identified as Coleoptera (blue), Psocoptera (red), and Trombidiformes (green) are indicated. Distinction is also made between sequences that correctly matched morphology based on a BLAST comparison to public *COI* databases (outlined), and those that did not match morphology (filled in). Individuals producing a single sequence are depicted as circles, whereas multiple sequences from the same individuals are depicted with triangles.

Individuals from the three arthropod orders with the lowest percentage of matching Illumina MiSeq sequences to morphology were selected for further analysis. Coleoptera, Psocoptera, and Trombidiformes had the highest percentages of non-matching Illumina MiSeq sequences when compared to the morphological identification (38.3%, 72.9%, and 98.2% respectively) (Fig. 2). All unique sequences produced by Illumina MiSeq (n = 1,211) were used for a Neighbor-Joining analysis based on pairwise distance (Fig. 3). Sequences recovered from Coleoptera, Psocoptera, and Trombidiformes were labeled as either matching or non-matching. Distinction was also made between individuals producing a single Illumina MiSeq sequence and individuals producing multiple sequences. For sequences recovered from individuals identified morphologically as Coleoptera, all but eight were contained within a single cluster including all matching sequences. The same case was true for Psocoptera, with only six sequences excluded, and Trombidiformes, with only one sequence excluded.

Sequences derived from individuals of Coleoptera, Psocoptera, and Trombidiformes that were contained within the correct order cluster but had a BLAST match to an incorrect order represent accurate DNA sequences generated via Illumina MiSeq sequencing that do not have a similar match within public *COI* databases. These sequences represent individuals for whom there is no close match in existing public *COI* databases and they couldn't be sequenced by Sanger sequencing. By using a similarity-based clustering approach it is possible to determine that most of the ‘failures’ of Illumina MiSeq sequencing were likely to be correct *COI* sequences. The revised Illumina MiSeq sequencing success rate for Coleoptera, Psocoptera, and Trombidiformes could be recalculated as 95.1%, 93.8%, and 98.2% respectively.

**Figure 4:**
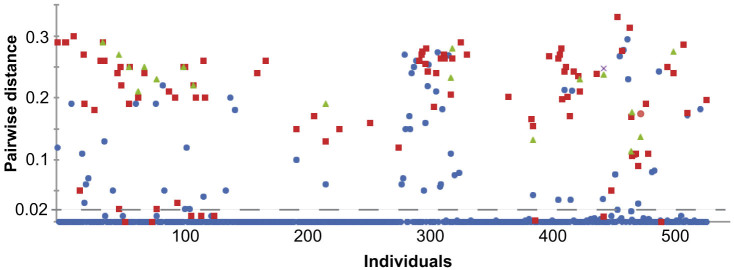
Pairwise distances between *COI* DNA sequences generated by Sanger-sequencing and Illumina MiSeq sequencing for 521 individual arthropods. Circles represent the first Illumina generated cluster, most similar to the Sanger, with other symbols representing second, third, and fourth Illumina sequences generated from the same individual. The area below the dashed line represents all Illumina sequences sharing at least 98% sequence similarity with a corresponding Sanger sequence from the same individual.

To investigate the accuracy of the Illumina barcoding approach as compared to Sanger sequencing, pairwise distances between Sanger and Illumina sequences generated by the same individual were calculated (Fig. 4). Of the 521 individuals for which both Illumina and Sanger sequences were produced, 429 (82%) produced Sanger and Illumina sequences with no sequence difference. A total of 463 (89%) individuals produced Sanger and Illumina sequences with less than 2% sequence difference.

To explore the sequencing depth of the Illumina MiSeq approach, all generated sequences from individuals of the two arthropod orders represented by the greatest number of individuals (Diptera n = 231; Hymenoptera n = 226), regardless of sequencing abundance, were recovered and analyzed. All sequences that were generated for each individual for the two *COI* segments were paired, dereplicated, and identified via BLAST comparison to public *COI* databases. Eleven individuals of Diptera and nineteen individuals of Hymenoptera generated at least one additional sequence that was identified as *Wolbachia* sp. (Proteobacteria: Rickettsiales: Anaplasmataceae).

## Discussion

It has been demonstrated that when DNA barcode libraries are more complete at the species level, the frequency of correct assignment of novel DNA sequences to upper taxonomic levels increases^29^,^30^. Large-scale efforts to recover DNA sequence data from fresh and archival specimens have shown some level of success (e.g., 50–86%) of potential DNA barcodes recovered^31^,^32^, but require a substantial amount of repeated sequencing effort.

A high Sanger sequencing failure rate is not unusual for large-scale DNA barcoding projects^26^. This is presumably due to insufficient amplification primer specificity, co-amplification of non-target amplicons, or the presence of competing sequence information (e.g., heteroplasmy and endosymbiotic bacteria) within individuals. Depending on the importance of the samples, some failures could be dealt with by using alternative PCR primers or changing the conditions of PCR prior to Sanger sequencing. In our present research, the low frequency of taxonomic assignment for *COI* sequences in some arthropod groups, is likely due to an underrepresentation of Costa Rican specimens in publicly available DNA barcode libraries^19^,^26^.

Our method, employing Illumina MiSeq sequencing platform to sequence individuals in parallel and to repetitively sequence from one individual in parallel, was able to recover DNA sequences from over 97% of specimens in a single attempt. This emphasizes the sensitivity of Illumina Miseq sequencing in recovering DNA barcodes from amplicons of low quality and/or quantity that cannot be equaled with Sanger sequencing. For 89% of these individuals, the Illumina sequences recovered share over 98% sequence similarity with the Sanger sequences recovered from the same individual. For the other 11% of individuals, it cannot be assumed that the Sanger sequence is ‘correct’ and the Illumina sequence ‘incorrect.’ These individuals possibly represent instances in which Illumina sequencing was able to recover an accurate sequence, whereas Sanger sequencing did not. The Illumina-generated barcode sequence of each individual is the product of over one thousand forward and reverse sequences of the first fragment and over one thousand forward and reverse sequences of the second fragment followed by assembling a contig of both fragment clusters. Conversely, the Sanger-generated sequence is the product of a single forward and a single reverse sequence. Sequencing error or sequence interpretation error can be detected and filtered out when thousands of sequences are considered, but not when only a single sequence is present.

In cases of multiple sequences being recovered from a single specimen, two different similarity-based assessments were used to distinguish the ‘true’ DNA barcode from intra-sample contamination (Fig. 1C and Fig. 3). In addition to public database comparisons, sequence similarity-based confirmation of recovered sequences may be necessary for some groups of organisms. This is especially true for groups like those arthropods for which there is low coverage in public DNA databases^19^.

The use of an HTS approach for building sequencing libraries allows for deep-sequencing to recover low-abundance sequences within each individual. These additional sequences can include heteroplasmous copies of the target gene and intracellular parasitic bacteria (e.g., *Wolbachia*), if present^33^.

**Figure 5:**
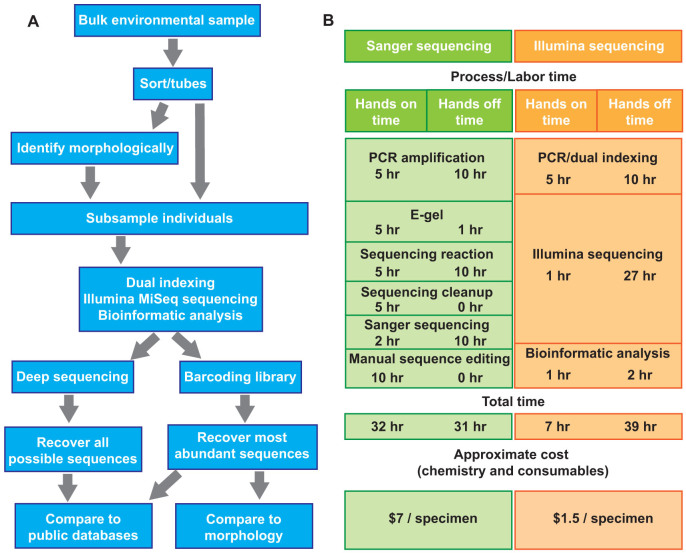
Workflow and cost and time analysis for the generation of DNA sequence data from multiple specimens using Illumina MiSeq sequencing. (A) The recommended new workflow. (B) A cost and time analysis of the new workflow versus conventional Sanger sequencing for ~1000 individuals.

We recommend a new workflow for generating DNA barcode sequences (Fig. 5A). Morphological identification of specimens is optional within the workflow and could be completed at a later time for confirmatory purposes. The method is adaptable to all organisms (i.e., plants, animals, fungi, bacteria) and all genetic markers (i.e., *COI*, *ITS*, *rbcL*, 16S, 18S). We calculated the cost and time investments in DNA sequence generation using Sanger sequencing compared to our new method (Fig. 5B). The new method represents a 27% reduction in total time and 78% reduction in hands-on time in addition to a 79% reduction in laboratory costs. This cost reduction will increase with projected advances in HTS technology. The presented workflow also allows research laboratories to employ a single HTS platform for both metabarcoding of bulk environmental samples and the generation of barcodes for individual specimens.

## Methods

The Malaise sample was collected at Bosque Humedo, Area de Conservación Guanacaste (latitude 10.85145; longitude -85.60801; altitude 290 m; date January 24–31, 2011). The sample was collected directly into 95% ethanol, and frozen at −20°C until thawed and processed in September 2011.

Tissue subsampled plates were DNA extracted using a Nucleospin Tissue kit (Macherey- Nagel Inc., Bethlehem, PA, USA) according to manufacturer's protocols. The standard 5′ end of the *COI* region was amplified using the primers LCO1490 and HCO2198^27^. Each PCR amplification contained 2 μL DNA template, 17.5 μL molecular biology grade water, 2.5 μL 10X reaction buffer, 1 μL 50X MgCl_2_ (50 mM), 0.5 μL dNTPs mix (10 mM), 0.5 μL forward primer (10 mM), 0.5 μL reverse primer (10 mM), and 0.5 μL Invitrogen Platinum Taq polymerase (5 U/μL) in a total volume of 25 μL. PCR conditions were 95°C for 5 minutes; 35 cycles of 94°C for 40 seconds, 51°C for 1 minute, and 72°C for 30 seconds; and 72°C for 5 minutes. Amplicon sequences were obtained using an ABI 3730XL sequencer (Applied Biosystems) and the sequencing traces were edited and assembled using CodonCode Aligner v 3.7.1.1 (CodonCode). The same region of *COI* was amplified for all individual DNA templates in two smaller, overlapping fragments (FC and BR) using Ill_LCO1490 x Ill_C_R (5′. GGIGGRTAIACIGTTCAICC.3′) and Ill_B_F (5′. CCIGAYATRGCITTYCCICG.3′) x Ill_HCO2198 primer sets respectively. The two fragments overlap by 82 bp. The above mentioned amplification regime was used with a modification in the annealing temperature (48°C for FC and 46°C for BR). All amplifications were completed on a Mastercycler ep gradient S (Eppendorf, Mississauga, ON, Canada). A negative control reaction with no DNA template was included in all experiments. The generated amplicons were dual indexed with unique 5-mer multiple identifiers (MIDs) from both directions. The designed MIDs include 40 different 5-mer identifiers (AAGCT, ATTGC, AGATC, AGCAT, TTCAG, TGATC, TCAAG, TGAGC, CAATG, CATTG, CTTGA, CTGAA, ATGCA, AGCTT, TGCAA, TGCCA, TCATG, CATGA, CTGAT, CATGC for FC fragment and ATGCT, ATGCC, AGCTG, AGCTC, TGCAT, TGCAG, TCAGA, TCAGG, CAGAT, CCTGA, CTCAG, CTGCA, ATCAG, AGCCT, ATCTG, TCAGC, TCTGA, TCCAG, CAGCT, CTGAG for BR fragment) The generated 22 amplicons plates were re-dual indexed and pooled into a single tube and sequenced on half of a Miseq flowcell using a V3 Miseq sequencing kit (300 × 2)(FC-131-1002 and MS-102-3001).

For all eleven plates, a total of 18,873,718 Illumina paired-end reads were filtered for quality and length. For each plate, the raw paired-end reads for the FC fragment and, separately for the BR fragment, were merged with SEQPREP software (https://github.com/jstjohn/SeqPrep) requiring a minimum Phred quality score of 20 and a minimum overlap of 25 bp. A total of 9,652,825 paired FC reads (mean 877,530 paired reads per plate) and a total of 6,020,424 paired BR reads (mean 547,311 paired reads per plate) were retained for further processing. Paired FC and paired BR reads were quality trimmed using CUTADAPT v1.4.1 requiring a minimum length of 300 bp and a maximum length of 400 bp for the FC fragment and a minimum length of 400 bp and a maximum length of 500 bp for the BR fragment^34^. A bioinformatic pipeline was created using Perl to dereplicate quality trimmed reads using CD-HIT v4.6 with the 'cd-hit-est' algorithm, and perform chimera filtering using USEARCH v6.0.307 with the '*de novo* UCHIME' algorithm^35^,^36^,^37^. At each step, cluster sizes were retained, singletons were retained, and only putatively non-chimeric reads were retained for further processing. A semi-automated bioinformatic pipeline was created using Perl to process the putatively non-chimeric FC and BR reads for each specimen and remove the associated tag and primers from each fragment. USEARCH with the UCLUST algorithm was used to de-replicate and cluster the remaining sequences using a 99% sequence similarity cutoff. A mapping file of tags was created and used to map FC and BR sequence clusters from each 96-well plate. A semi-automated bioinformatic pipeline was created using Perl to compare FC and BR fragment sequence clusters for each specimen using BLAST (blastn, megablast) requiring a minimum 98% sequence identity for each high-scoring segment pair (HSP), a minimum HSP length of 25 bp, with no more than 2 alignment mismatches. For BLAST results that meet these criteria, each full-length FC and BR fragment was paired using SEQPREP requiring a minimum of 80 bp overlap and a maximum of 0.02 mismatches allowed in the overlap region.

More details of the dual-indexing, amplification, sequencing and post sequencing bioinformatic processing are available by request from the corresponding author.

## Author Contributions

S.S. and M.H. conceived and designed the experiments. D.H.J. and W.H. collected all specimens examined. S.S. performed Sanger sequencing and Illumina MiSeq sequencing. S.S., J.F.G., T.M.P., B.G., R.D. and M.H. analyzed sequence data. S.S., J.F.G., D.H.J., W.H. and M.H. wrote and edited the final manuscript.

## Additional information

**Accession codes:** All Sanger and Illumina generated sequences have been deposited in GenBank (accession nos. KP843909-KP844445) and DRYAD (doi:10.5061/dryad.j897m).
